# State Liability for Failure to Control the COVID-19 Epidemic: International and Dutch Law

**DOI:** 10.1017/err.2020.21

**Published:** 2020-04-02

**Authors:** Lucas BERGKAMP

**Affiliations:** * Attorney-at-Law, Brussels, Belgium; email: lbergkamp@hunton.com.

## I. Introduction

The COVID-19 epidemic has caused governments in Europe to impose a variety of measures to fight the spread of the disease. Some governments have adopted relatively relaxed measures or adopted strict measures late, while some have been more proactive and implemented restrictions early on.

This article discusses the potential liability of governments in relation to neglicence and omissions with respect to COVID-19 measures. The focus is on China and The Netherlands. State liability can arise if governments have been negligent in addressing the threat of the COVID-19 epidemic, specifically where they have created risks due to not implementing restrictions or not doing so in a timely manner, or otherwise have failed to protect public health and human lives. These issues are analysed with reference to international law and the laws of The Netherlands, which has a well-developed – albeit idiosyncratic – system of state liability.^1^

Of course, it is also possible that governments are liable for damages caused by measures to fight COVID-19. For example, regulations requiring the closure of cafes and restaurants will cause economic harm to the operators thereof. This kind of potential liability is not discussed in this article. It should be noted, however, that governments are likely to offer compensation for the damages caused by these measures.

## II. International state liability

In addition to liability under national law, a state could be liable under international state responsibility. The International Law Commission has adopted a set of rules for “responsibility of states for internationally wrongful acts”.^2^ A state commits an “internationally wrongful act” when its action or omission: (1) is attributable to the state under international law; and (2) constitutes a breach of an international obligation of the state.

Given the all-encompassing character of the state’s role in combatting epidemics, an omission in this respect is likely to be attributable to a state. Under international law, states have a duty to cooperate with other states and to protect other states against harmful acts by individuals from within its jurisdiction.^3^ Arguably, a reporting obligation for infectious disease outbreaks^4^ can be inferred from the duty of state cooperation and the World Health Organization’s (WHO) International Health Regulations.^5^

The question is whether China has met its international law obligation in relation to the COVID-19 outbreak in Wuhan. It has been argued that China has “suppressed information about the [corona]virus, done little to contain it, and allowed it to spread unchecked in the crucial early days and weeks”.^6^ Reportedly, the Chinese authorities waited seven weeks to institute a lockdown in Wuhan, despite knowledge of the virus’s spread; by then, some five million people had already left Wuhan.

China waited until 31 December 2019 before reporting “a pneumonia of unknown cause detected in Wuhan, China” to the WHO Country Office.^7^ Earlier, the doctor who blew the whistle on the outbreak in Wuhan had been silenced by the Chinese authorities.^8^ On 15 January 2020, China reported that it had not found evidence of human-to-human transmission of the coronavirus.^9^ In addition, there is evidence that the Chinese government, despite the SARS outbreak, failed to adequately regulate wildlife markets, which are major sources of viruses, including coronaviruses.^10^

Thus, China may well be liable under international law based on a “wrongful act”. If so, China is required to “make full reparation for the injury caused by the internationally wrongful act”. Provisions of Chinese law cannot justify an internationally a wrongful act. The term “injury” is defined to include “any damage, whether material or moral, caused by the internationally wrongful act of a State”.^11^ Economic damage, lost profits and “any financially assessable damage”, are covered as well.^12^ Hence, China’s scope of state liability under international law is broad.

Of course, China is not the only state that is exposed to international state responsibility; other states that have failed to meet their obligations vis-à-vis other states may be liable, too. For instance, it has been reported that Austrian ski resorts ignored COVID-19 outbreaks in order to avoid harm to their economies.^13^ Such omissions by municipal or local governments are likely attributable to the state of Austria, and thus will entail Austria’s state liability. The Netherlands, too, may be exposed to international state responsibility, although, unlike China, it may be able to invoke as a defence that the WHO also acted very late, due, in part, to its unwillingness to confront China.^14^ Whether any of these states, in fact, will be held liable under international law is a political decision to be made by the states that suffered harm.

## III. The Dutch government’s response to the COVID-19 outbreak

The response of the Dutch government to the COVID-19 pandemic has been slow. On 29 January 2020, a member of the Parliament’s Second Chamber requested an urgent debate on the COVID-19 outbreak, but his request was dismissed by the majority.^15^ One day later, on 30 January 2020, the WHO declared the COVID-19 outbreak a “Public Health Emergency of International Concern”.^16^ This followed a call by the WHO on 13 January 2020, after the first COVID-19 patient outside of China had been confirmed, for active monitoring and preparedness. On 21 February 2020, the WHO warned that “the window of opportunity to contain the outbreak is narrowing and that the international community needs to act quickly”. To assist countries in preparing, the WHO published a checklist, which includes questions such as whether there are enough medical supplies.^17^

In relation to public health, the government of The Netherlands is assisted by the State Institute for Public Health and the Environment (“RIVM”), which is part of the Ministry of Public Health, Well-being and Sports.^18^ In the early days of the COVID-19 epidemic, the RIVM was effectively speaking on behalf of the Dutch government. As discussed below, the RIVM’s communications caused much confusion, and some of them were incorrect, not supported by science or inconsistent with the WHO’s recommendations.^19^

With things spinning out of control, the Cabinet, led by Prime Minister Mark Rutte, had to get up to speed on the issues and took over some of the communications in relation to the policy measures. Three problems with the Dutch government’s response to the COVID-19 outbreak are discussed below: (1) the objective pursued by the Dutch government in addressing the COVID-19 epidemic; (2) the delay in taking adequate response measures (travel restrictions, restrictions on social interactions); and (3) the lack of preparedness and transparency in addressing the outbreak. Before doing so, the next section briefly discusses the liability of the state for inadequate policy.

## IV. Dutch state liability rules

There are two ways in which the liability of the Dutch government can be engaged by citizens of The Netherlands. First, a non-governmental organization (NGO) has a right to bring a class action against the government to force it to adopt more stringent policy or to seek compensation for damages caused by inadequate policy.^20^ A recent Supreme Court ruling in a climate policy-related case (Urgenda) has confirmed that such NGOs have broad standing rights, and that the right to life laid down in the European Convention on Human Rights imposes on the government a positive obligation to protect a wide range of health and security risks, including climate change-related risks.^21^ The court’s judgement also eased causation requirements by introducing the concept of partial responsibility, which is related to proportional causation.

Second, any victim of an unlawful act committed by the government may bring an action to seek compensation for damages.^22^ In both cases, the standard for liability is the same: the state commits an unlawful act if it, through commission or omission, infringes on citizens’ rights or disregards its duty under the law or social norms. To succeed, a victim must prove that there is a causal link between the state’s violation and the damage he suffered.^23^

## V. Objective pursued

In the early days of the COVID-19 breakout, the Dutch government’s objective was not the protection of public health; rather, it was the prevention of public concern so as to avoid economic disruption. The RIVM’s communications in January and February 2020 illustrate how the RIVM attempted to lull people into a false sense of security.^24^ Travel restrictions and other measures to limit social interactions were not imposed until very late. The overriding objective was avoiding any economic harm. The RIVM was in the driver’s seat.^25^

Once this objective became politically untenable, the new objective became creating “herd immunity”. This objective also proved to produce significant backlash, as people began to realize that this would result in a peak of many deaths, particularly among the elderly and vulnerable. In response, the Prime Minister attempted to reframe the objective as “controlled population immunity”. The current objective is unclear, but appears to be closer to minimization of COVID-19 deaths and suppression of the outbreak, although a week into the lockdown the political pressure to relax the measures is already building up.

## VI. Delay in response measures

As the initial objective was avoiding panic and economic disruption, in the first phase, the RIVM did not recommend any measures or restrictions, even though KLM operated flights to Wuhan.^26^ After Italy declared a state of emergency on 31 January 2020,^27^ the RIVM did not discourage Dutch residents from travelling to Lombardy. Consequently, the first case in The Netherlands, which was reported on 27 February 2020,^28^ involved a patient who had returned from Lombardy. Nevertheless, the RIVM did not see any problem with large groups vacationing and skiing in northern Italy; this made a group of vacationing students uncomfortable, and they decided on their own volition to return to The Netherlands early.^29^ Only on 13 March were flights from COVID-19 hotspots cancelled.^30^ Likewise, the Mardi Gras carnival festivities in the south of The Netherlands were not cancelled, turning Brabant into a hotspot of COVID-19 infections.^31^

Limitations on social interactions were also imposed very late and reluctantly. When Prime Minister Rutte announced the recommendation to refrain from handshakes, he shook the hand of the RIVM’s chief following the announcement.^32^ The government did not take serious measures to fight the epidemic until 16 March 2020, after the Federation of Medical Specialists raised the red flag^33^; the next day, the government decided that schools, childcare centres, bars, restaurants, sports clubs and brothels were required to close down.^34^ Further measures including sanctions, under the misnomer of “smart lockdown”, were adopted as late as 23 March 2020.^35^ Even then, much confusion over the measures remained.^36^

## VII. Lack of preparedness and transparency

On 3 February 2020, the RIVM announced that it is “very well prepared if the virus makes it to The Netherlands”.^37^ As the crisis unfolded, however, it became clear that the preparations were inadequate at various levels – there were insufficient testing, tracing and monitoring capabilities, there were not enough masks and protective clothing and equipment, the capacity of hospitals and intensive care units appeared to be inadequate,^38^ there was no system in place for the distribution of COVID-19 patients across hospitals and there was no system for population measurement of body temperature.^39^

During the entire process of fighting the COVID-19 epidemic, the Dutch government gave the impression of trying to catch up with developments. The failure to test the population has been a key issue. Consistently, the WHO has recommended testing as the core element of the strategy to fight the epidemic.^40^ The RIVM, however, restricted testing to only a few small groups; even healthcare professionals were not consistently tested. The north of The Netherlands, which had fewer COVID-19 cases, disagreed with the RIVM’s recommendations and announced a broad testing programme.^41^ It has also been argued that the RIVM’s data are outdated.^42^

Neither the RIVM nor the Dutch government has been transparent about the data and analysis underlying their recommendations and policy measures. No sound risk assessments, no models or scenarios and no cost–benefit analyses of alternative policy measures have been published. As discussed above, the objective of the COVID-19 policy has changed over time, and it is still unclear. In addition, neither a strategy nor an action plan was ever clearly articulated. The websites of the RIVM and the Ministry of Public Health provide only limited data and analysis; it is impossible to determine which data, analyses and assumptions the RIVM and the government have used to arrive at their forecasts and interventions. Public participation in the process is impossible. As a result of this mismatch and the resulting vacuum, in the initial phase, the RIVM, which has no democratic legitimacy, determined key policies, including the policy of preventing public concern and economic harm. After public outcry drew politicians into this policy-making process, most of the time, the RIVM continued to be in the driver’s seat, as the politicians preferred to hide behind the science.

As of 31 March 2020, there are 12,595 confirmed cases of COVID-19 in The Netherlands, 4712 patients have been hospitalized, 1070 have received intensive care^43^ and 1039 have died.^44^ Due to its tardy and inadequate response, the state of The Netherlands ranks in the upper tier in terms of COVID-19 deaths per million inhabitants.^45^

## VIII. Conclusions

The COVID-19 crisis raises questions around the role of governments in managing the epidemic. This article briefly reviewed the role of China and The Netherlands. In each case, negligent omissions and other careless or unlawful acts are triggers of potential state liability. In the case of China, its belated response and initial cover-up of the outbreak in Wuhan allowed the virus to spread beyond China. These omissions may well constitute an international wrong for which China is liable vis-à-vis other states that suffered damage, including economic damage, as a result thereof.

The Dutch government’s response to the COVID-19 epidemic has been inadequate in many respects. Consequently, under Dutch tort law, the state of The Netherlands may be liable for damage caused by these omissions. The recent Supreme Court ruling in the Urgenda climate case provides further support for these kinds of claims. Causation will likely be a key issue, but the concepts of partial responsibility and proportional causation may help here. If the state must protect its citizens against the remote risks of climate change in the second half of the century, they surely must protect them against the immediate risks of COVID-19 in 2020.^46^

In short, the COVID-19 epidemic may not be over when the infections have abated. States that have failed in controlling the spread of the virus are likely to be held accountable and, possibly, liable.

## Footnotes

1 The civil courts in The Netherlands also handle claims against the state in relation to policy matters.

2 ILC, Responsibility of States for Internationally Wrongful Acts (2001).

3 See, eg, Trail Smelter case (United States v. Canada), *Reports of International Arbitral Awards*, 16 April 1938 and 11 March 1941, Vol III, pp 1905–98.

4 O Bozhenko, “More on Public International Law and Infectious Diseases: Foundations of the Obligation to Report Epidemic Outbreaks”, *EJIL:Talk!*, 15 August 2019 <https://www.ejiltalk.org/more-on-public-international-law-and-infectious-diseases-foundations-of-the-obligation-to-report-epidemic-outbreaks>.

5
World Health Organization, International Health Regulations (2nd edn, Geneva, WHO Press 2005).

6 S Hamid, “China Is Avoiding Blame by Trolling the World”, The Atlantic, 19 March 2020 <https://www.theatlantic.com/ideas/archive/2020/03/china-trolling-world-and-avoiding-blame/608332>.

7 WHO, “Coronavirus” <https://www.who.int/emergencies/diseases/novel-coronavirus-2019/events-as-they-happen>.

8 H Davidson, “Chinese Inquiry Exonerates Coronavirus Whistleblower Doctor”, *The Guardian*, 20 March 2020 <https://www.theguardian.com/world/2020/mar/20/chinese-inquiry-exonerates-coronavirus-whistleblower-doctor-li-wenliang>.

9 N Givas, “WHO Haunted by Old Tweet Saying China Found No Human Transmission of Coronavirus”, *New York Post*, 20 March 2020 <https://nypost.com/2020/03/20/who-haunted-by-old-tweet-saying-china-found-no-human-transmission-of-coronavirus>.

10 Vox, “How wildlife trade is linked to Corona viruses”, 6 March 2020 <https://www.youtube.com/watch?v=TPpoJGYlW54>.

11 ILC, Responsibility of States for Internationally Wrongful Acts (2001), supra, note 3, Art 31.

12
ibid, Art 36.

13 C Boyd, “How Austria’s ‘Ibiza of the Alps’ Ski Resort Helped Spread Coronavirus Throughout Europe as Officials Probe Whether Infections at the Resort Were Covered Up to Protect Tourism”, *Mail Online*, 24 March 2020 <https://www.dailymail.co.uk/news/article-8147199/Austrian-ski-resort-probed-claims-covered-coronavirus-cases.html>.

14 SL Miller, “Abolish the World Health Organization”, *Spectator USA*, 24 March 2020 <https://spectator.us/abolish-world-health-organization>; B Blanchard, “Taiwan Says WHO Not Sharing Coronavirus Information It Provides, Pressing Complaints”, *Reuters*, 30 March 2020 <https://www.reuters.com/article/us-health-coronavirus-taiwan-who-idUSKBN21H1AU>.

15 B Reijmerink, “FLASHBACK: VVD lachte Thierry Baudet UIT toen hij corona-alarm sloeg!” *DDS*, 23 March 2020 <https://www.dagelijksestandaard.nl/2020/03/flashback-vvd-lachte-thierry-baudet-uit-toen-hij-corona-alarm-sloeg>.

16 WHO, “Coronavirus”, supra, note 8.

17
ibid.

18 Rijksinstituut voor Volksgezondheid en Milieuhygiene <https://www.rivm.nl>.

19 L Gasthuis, “De coronacrisis in tweets van het RIVM: een tijdlijn”, *Elsevier Weekblad* <https://www.elsevierweekblad.nl/nederland/achtergrond/2020/03/tijdlijn-coronacrisis-in-tweets-rivm-743404>.

20 Art 3:305a, Burgerlijk wetboek.

21 Hoge Raad, 20 December 2019 <https://www.rechtspraak.nl/Bekende-rechtszaken/klimaatzaak-urgenda>.

22 Art 6:162, Burgerlijk wetboek.

23 Cf L Di Bella , De toepassing van de vereisten van causaliteit, relativiteit en toerekening bij de onrechtmatige overheidsdaad (Rijksuniversiteit Leiden, 2014 ).

24 “The risk of a COVID-19 patient in The Netherlands is small” (21 January 2020); “the disease does not appear to be very contagious” (24 January 2020); “it is possible that the virus reaches The Netherlands, but the chance that it will spread here is small” (28 January 2020); if the virus arrives in The Netherlands, “various measures will be effected that will prevent the virus from spreading” (3 February 2020); “only sick people are contagious” (11 February 2020); the RIVM explains in a video why people should not be worried (14 February 2020); “Dutch hospitals can provide the right care and intensive care units have sufficient capacity” (24 February 2020); “the virus is quite like influenza” (25 February 2020); schools do not have to be closed (1 March 2020); there is no reason to cancel mass events and concerts (5 March 2020); and “the mortality rate is not disturbing” (12 March 2020). L Gasthuis, “De coronacrisis in tweets van het RIVM: een tijdlijn”, supra, note 20.

25 L Bergkamp, “Corona-pandemie: economische technocratie faalt in tijden van crisis – De feiten rond COVID-19 liegen niet”, *OpinieZ*, 15 March 2020 <https://opiniez.com/2020/03/15/corona-pandemie-economische-technocratie-faalt-in-tijden-van-crisis/lucas-bergkamp>.

26 KLM, <https://www.klm.com/en-be/flights-to-wuhan>.

27 O Schneider, “Coronavirus Update: Italy Declares State of Emergency”, *The Brussels Times*, 31 January 2020 <https://www.brusselstimes.com/all-news/92889/coronavirus-update-italy-declares-state-of-emergency>.

28 “Eerste geval van corona in Nederland, op slechts enkele kilometers van Belgische grens: ‘Vraag is nu: “Met wie is hij in contact geweest?”’”, *Nieuwsblad*, 27 Feberuary 2020 <https://www.nieuwsblad.be/cnt/dmf20200227_04868284>.

29 C Berkhout, “Vindicat breekt skitrip in Italië af: ‘We hoefden van de GGD niet eerder terug, maar we willen de zorgen over coronavirus wegnemen’”, *Dagblad van het Noorden*, 5 March 2020 <https://www.dvhn.nl/groningen/Vindicat-breek-skireis-eerder-af-We-hoefden-van-de-GGD-niet-eerder-terug-maar-we-willen-de-zorgen-wegnemen-25425081.html>.

30 J Bailey, “Netherlands to Block Flights from Coronavirus Hotspots Later Today”, *Simple Flying*, 13 March 2020 <https://simpleflying.com/netherland-to-block-flights-from-coronavirus-hotspots-later-today>.

31 “RIVM, ‘Het lijkt er niet op dat carnaval in Tilburg probleem was’” <https://www.nu.nl/279332/video/rivm-het-lijkt-er-niet-op-dat-carnaval-in-tilburg-probleem-was.html>.

32 “Oeps: Rutte schudt hand na afkondigen handenschudverbod”, *RTL Nieuws*, 9 March 2020, <https://www.rtlnieuws.nl/nieuws/video/video/5050092/oeps-rutte-schudt-hand-na-afkondigen-handenschudverbod>.

33 “Medisch specialisten zetten vraagtekens bij openhouden scholen”, *Parool*, 14 March 2020 <https://www.parool.nl/nederland/medisch-specialisten-zetten-vraagtekens-bij-openhouden-scholen~b378b4ea>.

34 E Schaart, “Dutch PM: We Won’t Impose National Lockdown”, *Politico*, 16 March 2020 <https://www.politico.eu/article/dutch-pm-mark-rutte-we-wont-impose-national-lockdown-coronavirus-covid19>.

35 “The Dutch Ban Gatherings Until June 1, Give Mayors More Powers, Bring in Fines”, *Dutch News*, 23 March 2020, <https://www.dutchnews.nl/news/2020/03/the-dutch-ban-gatherings-to-june-1-give-mayors-more-powers-bring-in-fines>.

36 “Coronamaatregelen: wat mag wel en niet tot 6 april (en 1 juni)?”, *NOS*, 24 March 2020 <https://nos.nl/artikel/2328183-coronamaatregelen-wat-mag-wel-en-niet-tot-6-april-en-1-juni.html>.

37 L Gasthuis, “De coronacrisis in tweets van het RIVM: een tijdlijn”, supra, note 20.

38 The state ignored warnings in 2016 that there would not be sufficient intensive care beds in case of an epidemic: “Crisisteams waarschuwden al in 2016 voor tekort ic-bedden bij pandemie”, *nu.nl*, 27 March 2020, <https://www.nu.nl/coronavirus/6040662/crisisteams-waarschuwden-al-in-2016-voor-tekort-ic-bedden-bij-pandemie.html>. An order for additional respirators was placed only by mid-March: “Philips-topman: ‘Nederland bestelde beademingsapparaten twee weken geleden’”, *NOS*, 30 March 2020 <https://nos.nl/artikel/2328776-philips-topman-nederland-bestelde-beademingsapparaten-twee-weken-geleden.html>. The RIVM used an erroneous model to predict the number of intensive care beds required: B Soetenhorst and J van Kempen, “IC’s sneller vol: ‘RIVM heeft met verkeerde prognoses gerekend’, *Parool*, 30 March 2020 <https://www.parool.nl/nederland/ic-s-sneller-vol-rivm-heeft-met-verkeerde-prognoses-gerekend~baae999b>.

39 Several measures implemented by Singapore have not been implemented in The Netherlands: L Bergkamp, “Corona-pandemie: economische technocratie faalt in tijden van crisis”, supra, note 26.

40 “WHO head: ‘Our key message is: test, test, test’”, 16 March 2020 <https://www.bbc.com/news/av/world-51916707/who-head-our-key-message-is-test-test-test>.

41 “Groningen slaat het landelijk advies in de wind en gaat juist extra testen op corona, deze UMCG-viroloog legt uit waarom”, *Dagblad van het Noorden*, 23 March 2020 <https://www.dvhn.nl/groningen/Groningen-slaat-het-landelijk-advies-in-de-wind-en-gaat-juist-extra-testen-op-corona-deze-UMCG-viroloog-legt-uit-waarom-25490267.html>.

42 “Medische microbiologen: ‘Informatie RIVM loopt achter en is incompleet’”, *NOS*, 12 March 2020 <https://nos.nl/nieuwsuur/artikel/2326881-medische-microbiologen-informatie-rivm-loopt-achter-en-is-incompleet.html>.

43 Nationale Intensive Care Evaluatie, “COVID-19 infecties op de Nederlandse IC’s”, 31 March 2020 <https://www.stichting-nice.nl>.

44 RIVM <https://www.rivm.nl/nieuws/actuele-informatie-over-coronavirus>.

45 Worldometer <https://www.worldometers.info/coronavirus>.

46 Cf R Darwall, “The Coronavirus Pandemic versus the Climate Change Emergency”, *The Hill*, 29 March 2020 <https://thehill.com/opinion/energy-environment/490001-the-coronavirus-pandemic-versus-the-climate-change-emergency>.

